# Supplementary material for: Operando Characterization and Molecular Simulations Reveal the Growth Kinetics of Graphene on Liquid Copper During Chemical Vapor Deposition
Source: ACS Nano. 2024 Apr 30;18(19):12503–11. doi: 10.1021/acsnano.4c02070 (PMC11100484; doi:10.1021/acsnano.4c02070)
Supplement: Supplementary file 1 — nn4c02070_si_001.pdf [file nn4c02070_si_001.pdf]

Supporting Information

for

Operando Characterization and Molecular Simulations Reveal the Growth Kinetics of Graphene on Liquid Copper during Chemical Vapor Deposition

Valentina Rein[1,,‡], Hao Gao,[2,‡] Hendrik H. Heenen[2,*,‡] Wissal Sghaier[3], Anastasios C. Manikas[4], Christos Tsakonas[4], Mehdi Saedi[5,6], Johannes T. Margraf[2,7], Costas Galiotis[4], Gilles Renaud[3], Oleg V. Konovalov[1], Irene M.N. Groot[5], Karsten Reuter[2], Maciej Jankowski[1]*

1 – ESRF – The European Synchrotron, 71 Avenue des Martyrs, 38043 Grenoble, France
2 – Fritz-Haber-Institut der Max-Planck-Gesellschaft, Faradayweg 4–6, 14195 Berlin, Germany
3 – Univ. Grenoble Alpes and CEA, IRIG/ MEM/NRS, 38000 Grenoble, France
4 – FORTH/ICE-HT and Department of Chemical Engineering, University of Patras, 26504 Patras, Greece
5 – Leiden Institute of Chemistry, Leiden University, P.O. Box 9502, 2300 RA Leiden, The Netherlands
6 – Physics Department, Shahid Beheshti University, Evin, Tehran, 1983969411, Iran
7 – University of Bayreuth, Bavarian Center for Battery Technology (BayBatt), Weiherstraße 26, 95448, Bayreuth, Germany

*valentina.belova@esrf.fr
*heenen@fhi.mpg.de

Table of content

Introduction to CVD process……..S2
Role of hydrogen……S2
Quality control by Raman spectroscopy……..S3
Evolution of flake circumference and circularity……...S5
Evolution of the flake size with temperature and gas flow……S6
Energy of etching……...S8
Density Functional Theory Calculations……S8
Training of machine learning potentials……..S9
Free energy simulations……...S10
Validation of minimum……S14
Microkinetic model of competing carbon monomer and dimer attachment……..S16
References……S20

[‡]V.R., H.G., and H.H. contributed equally

Introduction to CVD process

In the graphene CVD process, a metal substrate surface, such as Cu, Ni, Pt, Fe, Ir, *etc*., acts as a catalyst for the decomposition of hydrocarbon precursor gas.[1] The elementary processes that occur during the CVD growth of graphene on either solid or liquid metal catalyst are schematically illustrated in Figure 1 of the main text. The catalyst substrate facilitates the chemisorption and dehydrogenation of precursor molecules such as methane, ethylene, or other hydrocarbons, resulting in carbon species such as monomers, dimers, *etc.*[2] The low solubility of C in Cu causes the formation of a 2D surface gas of diffusing C species rather than diffusion into the bulk. Nucleation occurs when the concentration of carbon species reaches a supersaturation level C_{nucl}. Nucleation can also be induced by impurities acting as seeds.[3] Additional C species then attach to the initial nuclei, forming flakes that grow in size (growth stage). Because the growth is a non-equilibrium process, it continues until the equilibrium concentration of carbon active species C_{eq} on the copper surface is reached, and the competing processes, *i.e.*, attachment and detachment, are balanced. In addition to surface diffusion (intralayer), C species can undergo interlayer diffusion when climbing up the graphene flake, thus overcoming a step-edge energy barrier.[4] There is also a continuing desorption of precursor atoms/molecules from the surface, which rate becomes significant at high temperatures as the sublimation of the metal substrate starts to play a role.

Role of hydrogen

The presence of hydrogen is vital in the CVD process.[5–9] On the one hand, it is assumed to participate in methane dehydrogenation and thus facilitate the formation of active C species, although a precise understanding of the detailed mechanism is still missing. On the other hand, H_2 etches the graphene, predominantly attacking defects and terraces above the first layer if there are any. Thus, to secure the growth of high-quality graphene, the partial pressure p_{H2} can be used to control the size and morphology (compact circular or hexagonal *vs.* dendritic/random shapes such as 'snowflakes' or 'flowers') of the islands and usually has to exceed many times the partial pressure of methane p_{CH4}.[5,7,10,11] If the concentration of the hydrocarbon precursor is insufficient, the etching process dominates, and the grown graphene flake is etched away.

Although growth at higher methane pressure cannot be followed with the same precision due to the high nucleation density and fast layer closure, we also explored the partial pressure range of H_2 between 0 (no H_2 flow) and the default value of 18.18 mbar (as in the base gas mixture of 200 sccm of Ar and 20 sccm of H_2) by using a 5% concentration of CH_4 in Ar with the highest flow of 75 sccm, the highest flow of H_2 of 20 sccm (the flows are limited by the setup), and the total flow in the range of 220–300 sccm (Figure S1). The growth rates reach the maximum around p_{H2} = 9.65 mbar, corresponding to a CH_4/H_2 ratio of 0.19, and then decline with the decrease of p_{H2} to zero. This bell-shaped dependence of the growth rates on p_{H2} is in good agreement with previous studies on solid substrates and confirms that the presence of H_2 is crucial in the CVD process.[8]

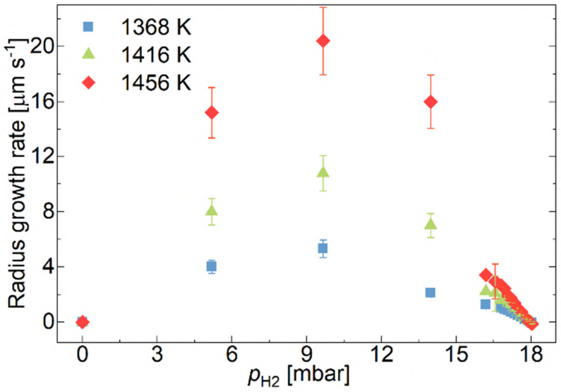

Figure S1. Lateral growth rates as a function of hydrogen pressure.

Quality control by Raman spectroscopy

Quality control of graphene samples grown on liquid Cu was performed *via* Raman spectroscopy. The samples grown at similar conditions described in the main text were transferred onto a Si/SiO_2 wafer through the wet transfer process[12] and subsequently examined under the Raman microscope. A commercial solution of poly methyl methacrylate (PMMA) in anisole (Microchem 495K) was diluted at 3 wt%. A thin PMMA film was spin-coated onto CVD graphene on Cu foil to form a homogeneous protecting layer on graphene. Afterwards, copper was etched by using a 0.15 M ammonium persulfate solution. The PMMA–graphene film was rinsed with distilled water and subsequently deposited onto a Si/SiO_2 substrate and dried at 50 °C under vacuum for 30 min to remove possible trapped water molecules. The PMMA was dissolved by acetone.

It is widely known that the Raman spectrum of graphene generally consists of three prominent peaks (Figure S2): G, D, and 2D.[13] The G peak (~1582 cm^{-1}) is present in all sp^2 carbon materials and corresponds to the in-plane doubly degenerate E_g phonon in the center of the Brillouin zone. The D peak is activated by the presence of structural defects (that do not include morphological defects like, *e.g.*, wrinkles) which lead to a double resonant process of transverse optical phonons around the K-point of the Brillouin zone (not seen in Figure S2). The 2D peak (second order of the D peak) arises from a double resonant 2-phonon process, which does not require the presence of defects. The characteristics of the D, G, and 2D peaks provide information about graphene, such as (1) the defect density through the ratio of intensities of the D and G peaks I_D/I_G, (2) the number of layers through the full-width-at-half-maxima (FWHM) and shape of the 2D peak and the I_{2D}/I_G ratio and, finally, (3) the residual stresses through the positions of the G and 2D peaks (Pos(G), Pos(2D)) and the FWHM of the 2D peak. Raman mapping was acquired from the Gr/SiO2/Si sample to examine the quality of the transferred graphene layer. An 80 x 25 μm^2 area was mapped with a step of 0.5 µm, and the results are presented in Figures S3 and S4.

The uniform distribution of the spectroscopic characteristics I_G and I_{2D} reveals that the sheet is continuous without cracks or tears in the examined area. The absence of the D peak, as seen from the representative spectrum in Figure S2, confirms the high quality of the produced graphene and the absence of structural defects. As demonstrated in our previous study,[14] a minimal D peak can be detected in our setup at the edges of small-sized flakes (~ 80 µm) in contrast to the larger flakes (~ 800 µm), which we ascribed to size/edge effects and not structural defects. In the present work, the size of the characterized flakes is in the range of hundreds of micrometers, and hence, no D peak is involved. The growth of monolayer graphene is confirmed from Raman spectra through the I_{2D}/I_G ratio of ~2.3 and the shape of the 2D peak, which is a single Lorentzian with FWHM around 29 cm^{-1}. Finally, the shift

of the G peak to higher wavenumbers (Figure S3) indicates the presence of minimal residual compressive biaxial stress, which originates from the contraction of Cu during cooling from elevated temperatures to room temperature, but also from the stresses that were applied to the graphene layer during the transfer process.

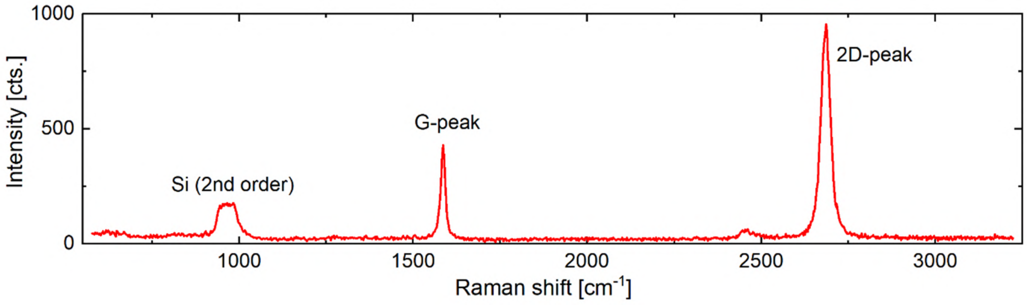

Figure S2. Representative Raman spectrum of graphene on liquid Cu, acquired with the 514 nm excitation source at room temperature after transfer on Si/SiO$_2$.

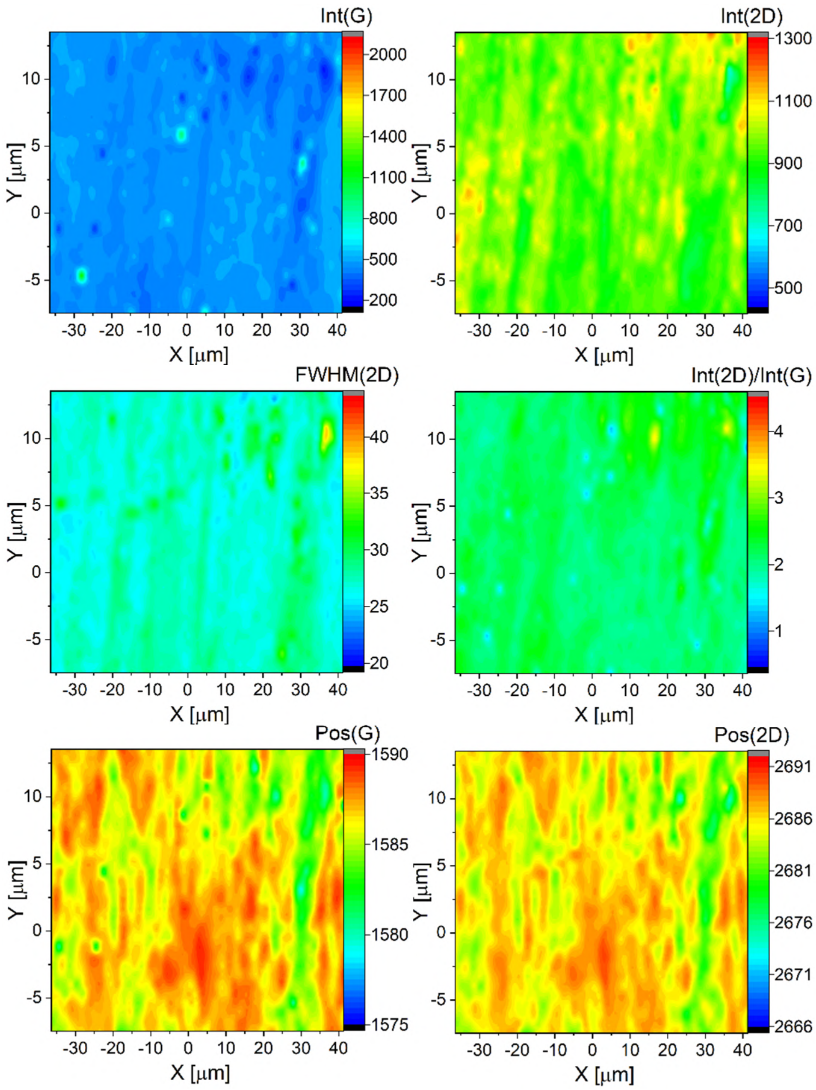

Figure S3. Raman mappings on the Gr/SiO$_2$/Si substrate acquired with a 514 nm excitation source after cooling to room temperature.

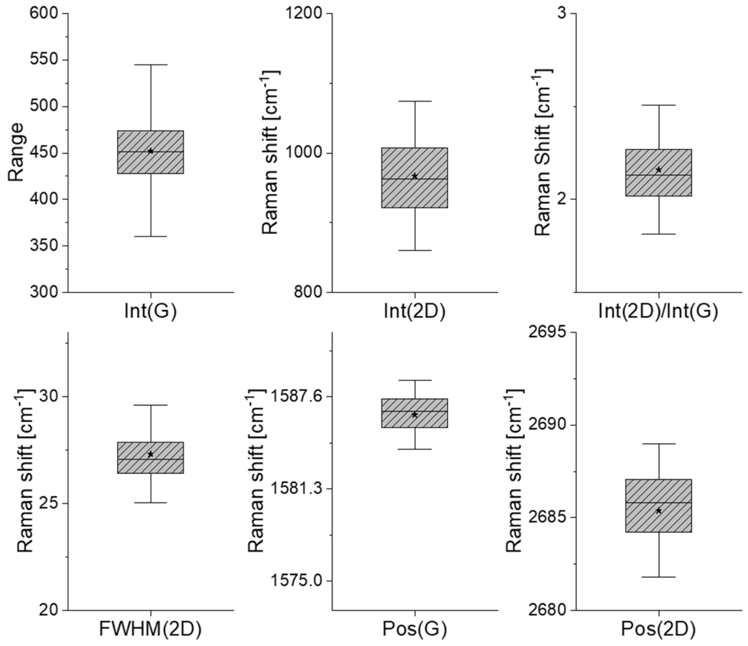

Figure S4. Statistical analysis in box plots corresponding to the Raman mapping in Fig. S3.

Evolution of flake circumference and circularity

The analysis of the flake circumference provides a good illustration of the morphology evolution depending on the methane pressure (Figure S5). Thus, for the four considered ranges of p_{CH4}/p_{H2} we see that the average circumference tends to follow the particular shapes: a rapid transition to a sharp concave dodecagon with a prominent external angle (10° and larger) at low methane pressure (Figure S5a); a slower transition from a hexagon to a concave dodecagon at the medium range (Figure S5b); a smooth transition from a circular shape to a hexagonal shape for the higher partial pressure ratio (Figure S5c); and the primarily circular shape with some minor deviations for the highest range (Figure S5d). A similar classification of the circularities of the corresponding set of flakes is provided in Figure S6.

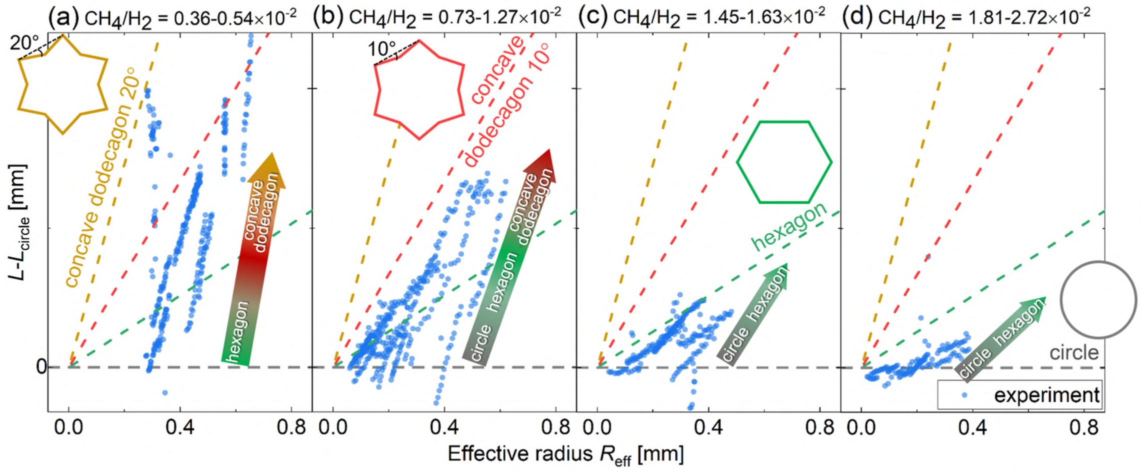

Figure S5. Graphene flake circumference L as a function of the flake size (effective radius) compared to an ideal circle (grey), ideal hexagon (green), an ideal concave dodecagon with an external angle of 10° (red), and 20° (dark yellow). The arrows indicate the shape transition with the flake size. The data is shown for different values of p_{CH4}/p_{H2}, which overall dominate the flake shape.

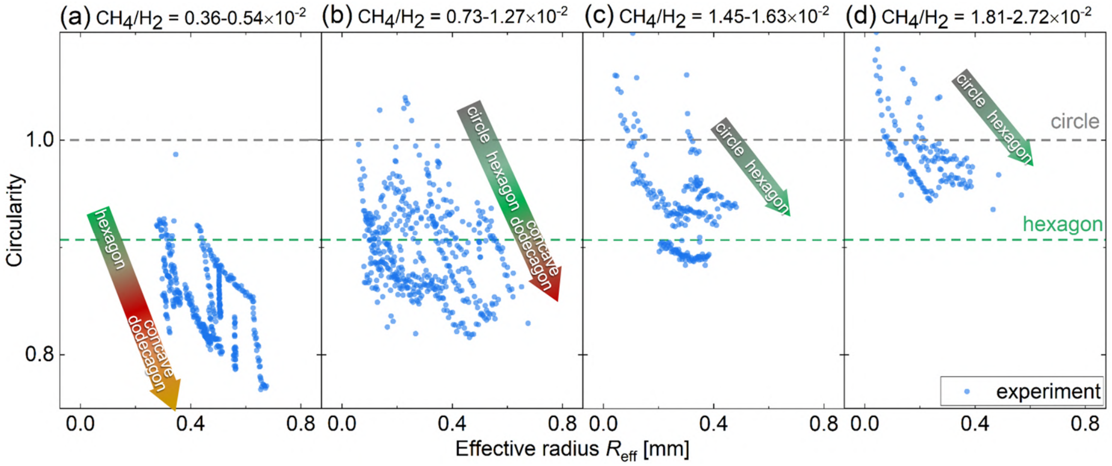

Figure S6. Flake circularity as a function of the flake size compared to an ideal hexagon (green) and an ideal circle (grey). The arrows indicate the shape transition with the flake size in accordance to Fig. S5. The circularity values above 1 are artifacts originating from the pixelation effect in the images. The data is shown for different values of p_{CH4}/p_{H2}, *which overall dominate the flake shape.*

Evolution of the flake size with temperature and gas flow

Figure S7 shows the time evolution of the effective flake radius $R_{eff} = 2A/L$ averaged over an array of flakes present in each recorded image frame for five growth temperatures and four groups of p_{CH4}/p_{H2}. The error is defined by the pixel size of 4 µm. In the plot, the time is reset to zero each time the methane flow changes. Therefore, the flakes at 'zero' time have the average size they reached during the previous experimental stage: either growth with a different rate or etching. After significant changes in the CH_4 flow, it may take up to tens of seconds to stabilize. Therefore, these points are excluded from the analysis. When the valve is open for the first time (bare surface without flakes), the time for flakes to be large enough to apply our software (size above ~15 µm) takes up to several minutes. Thus, the position of the datasets on the abscissa axis can be considered arbitrary, and the flake parameters have to be characterized by their change with time. The main observation is that the flake diagonal increases linearly with time for a given growth temperature, counting from the last change of methane flow. This observation is valid for a broad range of temperatures and partial pressures.

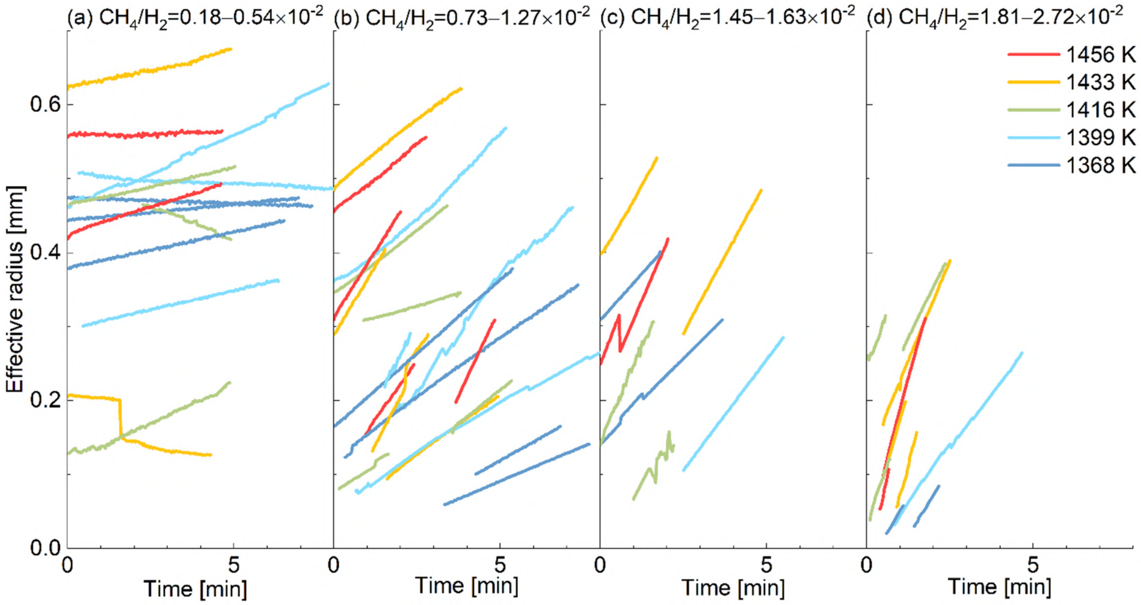

Figure S7. Evolution of the effective flakes' radius averaged over an array of flakes present in each image frame (the error is 4 μm) as a function of growth time for different growth temperatures and partial pressures. The time is set to 0 when the CH_4 flow is opened. The abrupt changes occur due to changes in the flakes' number in the region of interest as they float in and out of view during the experiment.

In previous studies, when CVD graphene growth on the solid copper catalyst was studied,[8,15,16] graphene growth rates were often defined as a change in area over time. However, we find here that the flake area grows proportionally to the square of time, which correlates directly with a linear radius evolution. Consequently, the areal growth rates are not constant with time and depend linearly on the flake size, as demonstrated in Figure S8. Using those in the Arrhenius equation may result in an inaccurate E_a value. Therefore, we choose the R_{eff} as the main parameter of the growth description.

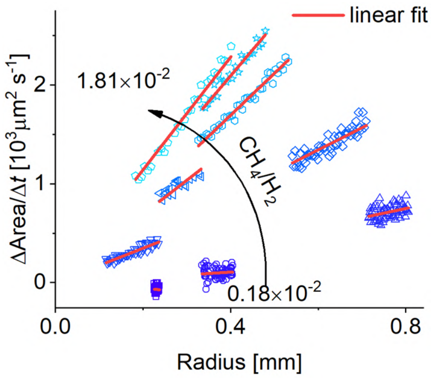

Figure S8. Areal flake growth rates in $\mu m^2\ s^{-1}$ as a function of the radius (shown for T = 1433 K).

It is also interesting to see whether the linearity of the lateral growth rate changes when strong interaction between the flakes emerges upon their coalescence. We consider this case in Figure S9 and Movie S2. Here, the nucleation was induced by a CH_4 'burst' in which CH_4 gas flowing at 12 sccm (2% CH_4 diluted in Ar) was accumulated for 20 seconds in the gas line before opening the valve to the reactor ('zero' time in Figure S9a). The p_{CH4}/p_{H2} stabilizes in a few seconds after the nucleation at the value of 1.27×10^{-2}, and it takes less than 80 seconds until all flakes in the region of interest merge. In Figure S9a, we track two parameters: the average flake diagonal (square symbols) and the average gap between flakes (circles). The gap starts to decrease (Figure S9a,b) shortly after the nucleation, then

becomes comparable with a flake size of 74 µm (Figure S9a,c) and reaches a minimum of 43 µm (Figure S9a,d). After this, the flakes with the minimal gap start to coalesce (top part in Figure S9d,e). The nominal average gap slightly increases because the merged flakes no longer participate in the data analysis. Despite the strong interaction between neighboring flakes, there is almost no noticeable deviation from the linear time dependence of the diagonal (accounted only for non-merged flakes) until all flakes have coalesced.

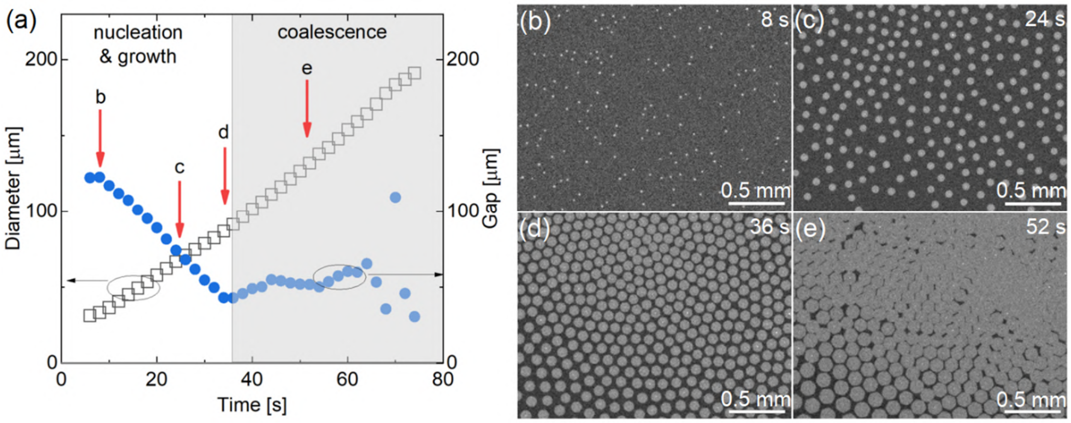

Figure S9. CVD growth on liquid Cu initiated by a CH_4' burst' when the CH_4 flow of 12 sccm (2% CH_4 diluted in Ar) was accumulated for 20 s in the gas line before opening the valve to the reactor. Then p_{CH4}/p_{H2} stabilizes at 1.27×10^{-2}: (a) average diameter (empty grey squares) and inter-flake gap (blue circles) evolution upon layer closing, the black arrows indicate the moments corresponding to the images in (b)-(e); (b)-(e) microscopy images of the different growth stages between nucleation and coalescence of a majority of the flakes as indicated by red arrows in (a). See also Movie S2.

Energy of etching

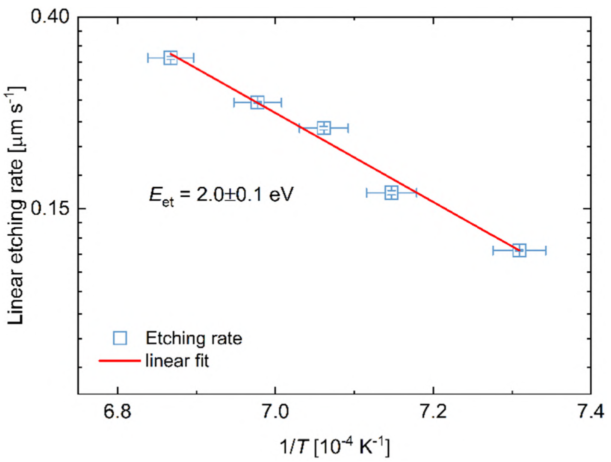

Figure S10. Arrhenius plot of the etching rates for p_{CH4} = 0. The fit gives E_{et} = 2.0±0.1 eV.

Density Functional Theory Calculations

All the first-principles calculations were performed by the full-potential, all-electron DFT package FHI-aims[17] using light default settings for the basis set and integration grids, the PBE[18] exchange-correlation functional, and an MBD correction[19]. K-point sampling was done with a density exceeding 60/Å k-points. Self-consistent electronic energies converged to within 10^{-6} eV.

Training of machine learning potentials

The machine learning (ML) potential was trained based on the Moment Tensor Potential (MTP) framework[20,21] with hyperparameters of level b = 20 and a cut-off of 6 Å. This MTP was trained to describe the adsorption of graphene on liquid Cu but did not include configurations relevant to the attachment of activated carbon intermediates from our previous work[22]. For this reason, we extend our previous MTP via an active learning approach, using the libatoms/wfl-framework,[23] as illustrated in Figure S11, where we iteratively perform variational enhanced sampling (VES)[24] simulations, including the attachment of carbon monomers and dimers to a zigzag edge as well as an armchair edge of graphene. After the different iterations, the MTP training and test set are updated and retrained. In each iteration, new configurations that are not well described by the current MTP are selected and added to the training or test set. These configurations are identified by similarity measure using the Smooth Overlap of Atomic Positions (SOAP) descriptor and selected using a furthest-point sampling (FPS) approach. This iterative active learning approach is repeated until the root mean square error (RMSE) converges. The number of configurations in the final training and test set is 197 and 80, respectively (see overview of configurations in Figure S12). The training and test force RMSEs of the final potential are 0.100 and 0.110 eV/Å, respectively, as shown in Figure S13.

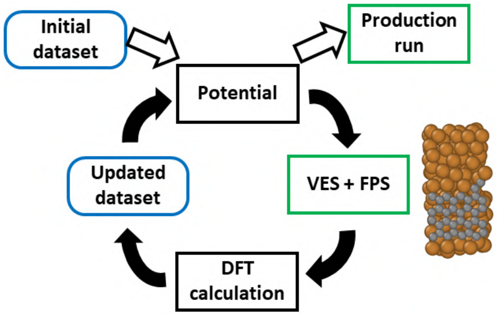

Figure S11. Framework of active learning. See the text for a detailed description.

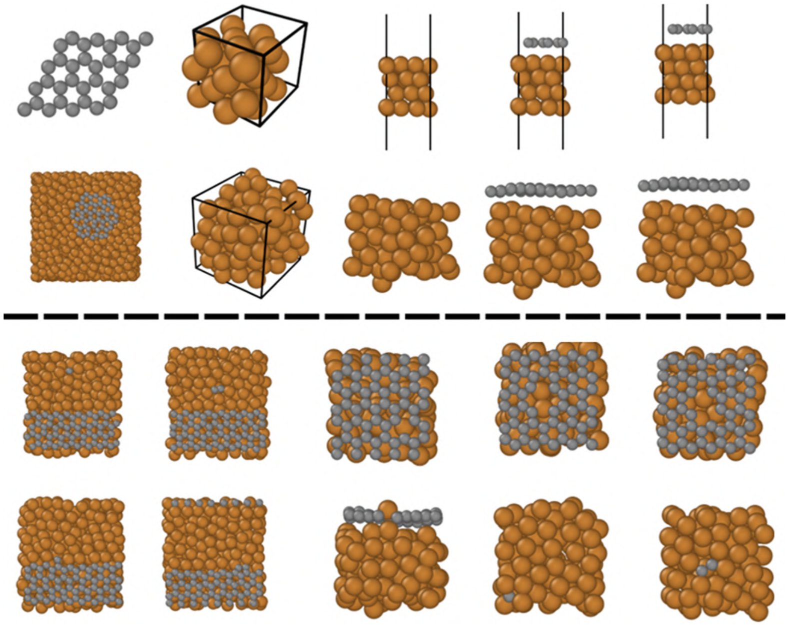

Figure S12. Representative configurations for training and testing, including those selected manually (top) and sampled by active learning (bottom). Grey are C atoms, and orange are Cu atoms.

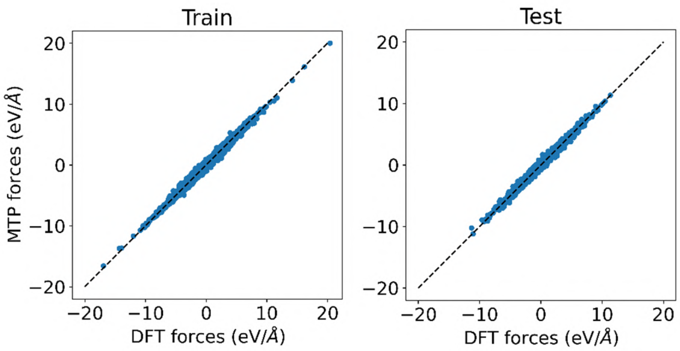

Figure S13. Comparison of forces predicted by MTP and DFT on both training and test sets enhanced by active learning.

Free energy simulations

Umbrella Sampling (US) simulations are conducted via PLUMED[25] in conjunction with LAMMPS[26]. We simulated free energy surfaces of the attachment of a carbon monomer or a dimer to graphene zigzag and armchair edges (mono-ZZ, dimer-ZZ, mono-AC, and dimer-AC) and the decomposition and formation of one carbon dimer from/to two monomers (mono-dimer). Five relative atomic models, mono-ZZ, dimer-ZZ, mono-AC, dimer-AC, and mono-dimer, are composed of $C_{385}Cu_{1213}$, $C_{386}Cu_{1213}$, $C_{337}Cu_{1213}$, $C_{338}Cu_{1213}$, and C_2Cu_{877}, respectively. In our models, we chose to simulate fully dehydrogenated carbon intermediates and graphene edges. The dehydrogenated state has previously been proposed to be as stable as hydrogenated edges,[27] its computational description requires a less complex MTP (fewer elements), and the simulated processes are less error-prone due to reduced configurational space.

For the US calculations, collective variables (CVs) must be defined to describe the reaction coordinate. As the CV for the attachment processes, we use the minimum distance between the carbon monomer/dimer and the graphene ribbon. For the decomposition of the carbon dimer, CV is defined as the interatomic distance between two carbon atoms. For each free-energy surface, the CV space is sliced into multiple narrow windows, and a biased molecular dynamics (MD) simulation is performed in each window. The simulations are propagated for 2 ns with a time step of 1 fs. A Nose-Hoover thermostat[28] with damping parameters of 0.1 ps for a temperature of 1370 K was employed to produce an NVT ensemble in all the narrow windows in CV space. The number of windows is 48 for mono-ZZ, dimer-ZZ, mono-AC, dimer-AC, and mono-dimer. We ensured for each window an even sampling around the bias following an unskewed normal distribution. The resulting free-energy surfaces are obtained from Umbrella Integration. For mono-ZZ/AC and dimer-ZZ/AC, carbon can be dissolved in liquid Cu. Graphene ribbons are placed on both sides of the liquid Cu slab to avoid unnecessary sampling of a clean liquid Cu surface. During dimer-ZZ/AC simulations, the carbon-carbon distance inside the dimer is limited to 3 Å to avoid the dissociation of the dimer. The convergence of barriers is shown in Figure S14 and S17; all the associated energy differences are displayed in Table S1. Sampling densities in cartesian space for mono-ZZ, dimer-ZZ, mono-AC, and dimer-AC are shown in Figure S15, and mono-dimer in Figure S17.

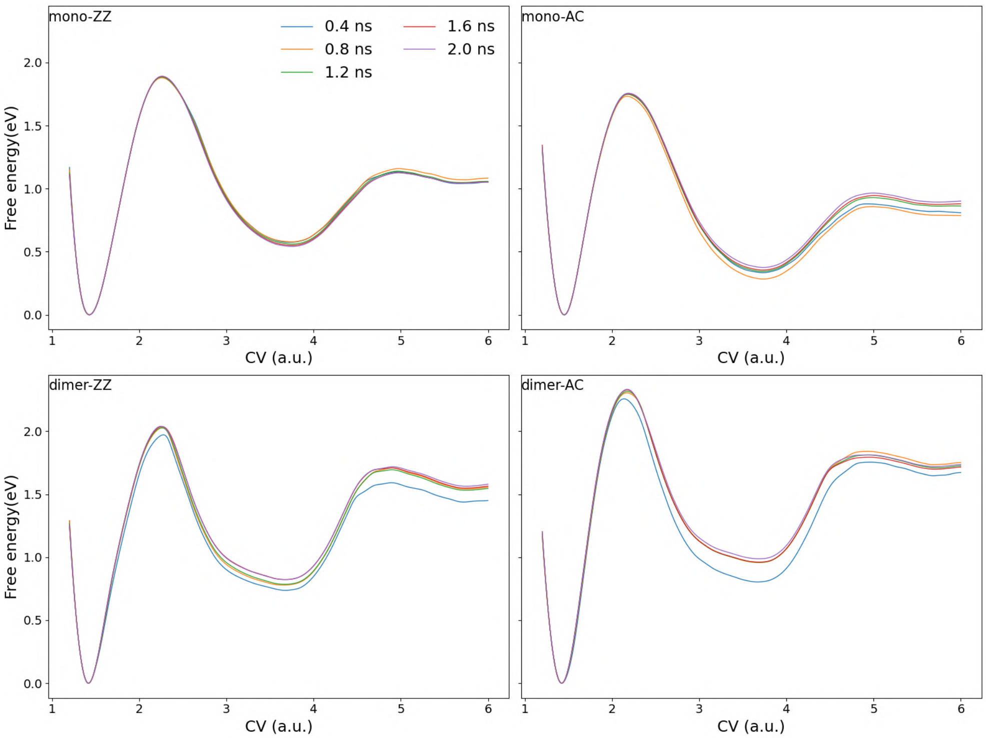

Figure S14. Free-energy surfaces of the attachment processes for different carbon species to different graphene edges. We display the energy profile at different simulation times for each window to show the simulations' convergence.

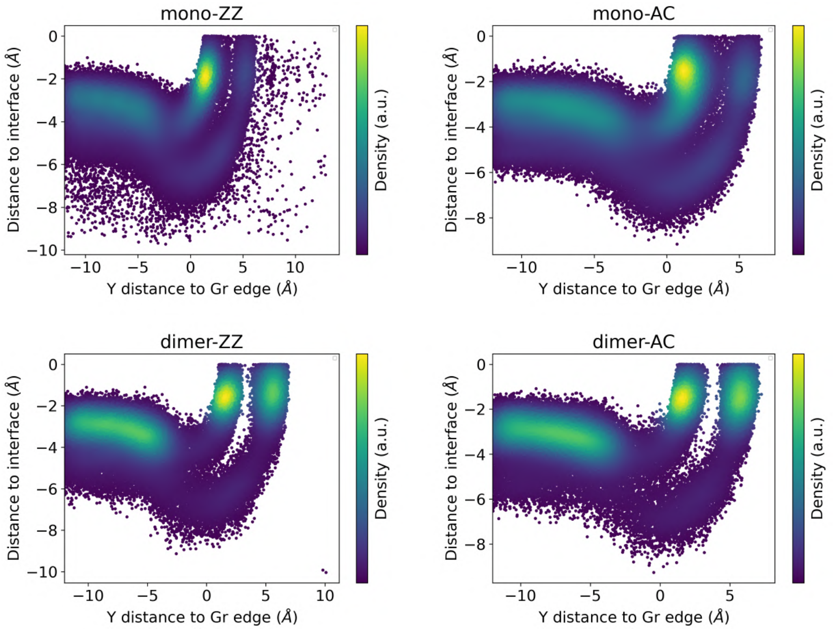

Figure S15. Sampling density for the four simulated attachment processes projected onto relative coordinates in cartesian space. The horizontal axis describes the distance between carbon species and the graphene edge along the normal direction. The negative value means carbon species are below the graphene ribbon, while the positive value means they are far from the graphene ribbon. The vertical axis describes the distance between the interface and carbon species. The negative value means carbon species are immersed in the liquid Cu. The colormap indicates the sampling density, where a high density indicates the location of the two minima (see main text).

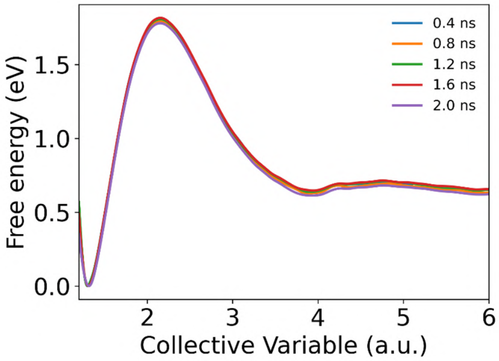

Figure S16. Free-energy surface of the dimer dissociation process. We display the energy profile at different simulation times for each window to show the simulations' convergence.

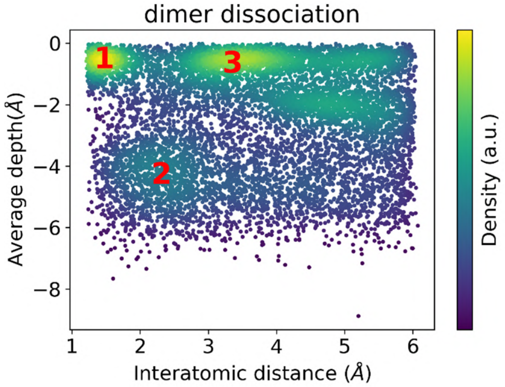

Figure S17. Sampling density for dimer dissociation processes projected from the CV onto relative coordinates in cartesian space. The horizontal axis describes the interatomic distance between two carbon atoms, and the vertical axis is the average depth of the two carbon atoms inside liquid Cu. The average depth is defined as the distance to the mean of the local surface atoms which are selected within a volume segment perpendicular to the Cu surface defined by a cut-off of 3 Å. The colormap illustrates high-density points that correspond to local minima on the free energy surface. It can be seen that the monomer and dimer (identifiable by long and short interatomic distances, respectively) are found both on the surface and in the bulk. It can be seen that the dimer is, to a larger degree, more surface-bound than the monomer. The bright regions in the plot are relative to different states marked numbers: 1 for dimer, 2 for transition state and 3 for monomer.

We elaborate on the details of the dimer attachment process to the ZZ edge (dimer-ZZ) by performing a refined simulation of the opening and closing of the 5-membered-ring (dimer-ZZ-open), which is the final state of the dimer-ZZ process. The corresponding CV is defined as the minimum distance between one atom in the dimer and the graphene ribbon, and the sampling range is limited to 3 Å so that another atom in the dimer remains attached to the graphene ribbon. The number of windows is 18 and the simulation time in each window is 1 ns, which was in this case sufficient for convergence. These results are shown in Figure S18 and Table S2.

To assess the finite size effect in this system, we also performed US for mono-ZZ with smaller ($C_{193}Cu_{877}$ with graphene ribbon width of ~11 Å) and larger ($C_{577}Cu_{1754}$ with graphene ribbon width of ~38 Å) cells (see Figure S19). Testing US simulations with the large cell indicates only a minor difference (0.02-0.03 eV) for attachment and detachment barriers (see Table S1 and S3), indicating no influence of the extended graphene sheet. In comparison, the difference in the attachment barrier between the small and the larger cells is more significant (0.07 eV), indicating a finite size effect for small graphene ribbons where the local minimum describing the detached state is likely less stabilized

due to a missing delocalization under the “short” graphene ribbon (see discussion "validation of minimum").

Table S1. Free-energy barriers for different carbon species and graphene edge.

Free energy barrier (eV)	Attachment/Association	Detachment/Dissociation
mono-ZZ	1.35	1.89
dimer-ZZ	1.22	2.04
mono-AC	1.38	1.75
dimer-AC	1.27	2.35
mono-dimer	1.17	1.78
mono-ZZ (small cell)	1.30	1.89
mono-ZZ (large cell)	1.37	1.86

Table S2. Free-energy barriers opening and closing process for the 5-ring on the zigzag edge.

Free energy barrier (eV)	Open	Close
dimer-ZZ-open	1.39	0.74

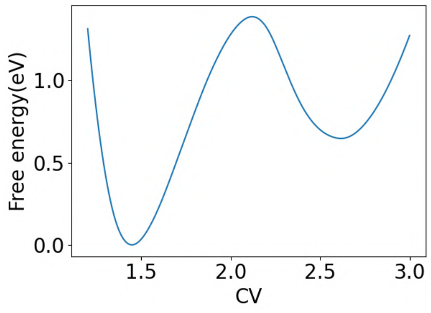

Figure S18. Free energy surface for dimer-ZZ-open.

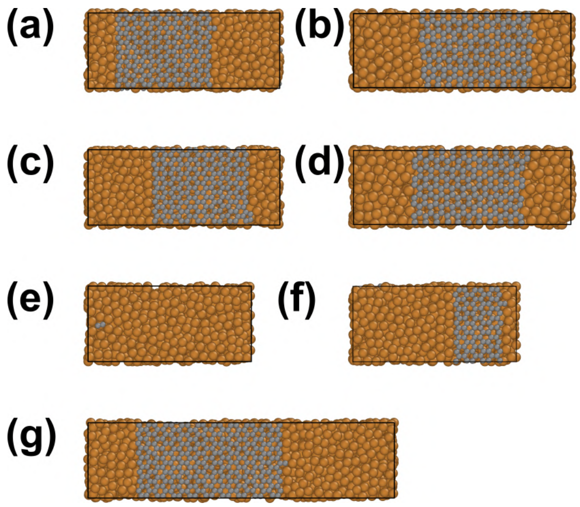

Figure S19 Simulation cells used for free energy calculations. (a) mono-ZZ; (b) mono-AC; (c) dimer-ZZ; (d) dimer-AC; (e) mono-dimer; (f) mono-ZZ (small cell); (g) mono-ZZ (large cell).

To extract meaningful free-energy differences and activation energies from the computed free-energy surfaces that are compatible with transition state theory, we follow the procedure suggested by Dietschreit *et al.*[29]. The activation energies are computed by $\Delta F^{\ddagger} = -k_B T ln \frac{\rho_{TS}\lambda_{TS}}{P_{RP}}$. Here, k_B is the

Boltzmann constant, T the temperature, ρ_{TS} is the probability density of transition states and P_{RP} is probability of reactant or product. Both densities can be obtained directly from the computed free-energy surfaces. For the transition state, the probability density is computed by $\rho_{TS} = e^{\frac{-F(\xi_{TS})}{k_B T}}$ where $F(\xi)$ is the free energy depending on CV computed by US and ξ_{TS} is the position of the transition state in CV space. For the reactant or product state, the probability is an intergral $P_{RP} = \int_{\Omega} d\xi\, e^{\frac{-F(\xi)}{k_B T}}$ over the region Ω of reactant or product in CV space. The $\lambda_{TS} = \left\langle \sqrt{\frac{h^2}{2\pi m_\xi k_B T}} \right\rangle_{TS}$ is the ensemble average of the de Broglie thermal wavelength of the quasiparticle relative to the CV (ξ) at transition states. The effective mass of the CV at the transition state is defined as $m_\xi^{-1} = \sum_{i=1}^{3N} \frac{1}{m_i} \left(\frac{\partial \xi}{\partial x_i}\right)^2$ where N is the total number of atoms. x_i and m_i run over all the Cartesian positions and atomic masses. In our simulations, all CVs are defined as the interatomic distance between two carbon atoms and the effective mass follows, therefore, as a constant: $m_\xi^{-1} = \frac{2}{m_{carbon}}$, leading to $\lambda_{TS} = 0.1925$ Å. We tested this procedure with different definitions of the CV for the same process (not shown), which leads to an alignment of otherwise different "uncorrected" reaction barriers and removes the bias derived from the choice of the CV. The corrected values are displayed in Table S3 and correspond to the barriers that govern the geometry of the investigated microscopic processes, determining the barriers relevant to the transition rates. We note that the corrections only lead to small changes in the data which also only leads to small quantitative differences in our microkinetic model (see below).

Table S3. Activation energies with and without correction.

Activation energy (eV)	Attachment or Dissociation or Closed (uncorrected/corrected)	Detachment or Dissociation or Open (uncorrected/corrected)
mono-ZZ	1.35/1.51	1.89/1.87
dimer-ZZ	1.22/1.38	2.04/1.99
mono-AC	1.38/1.54	1.75/1.72
dimer-AC	1.27/1.43	2.35/2.31
mono-dimer	1.17/1.44	1.78/1.73
dimer-ZZ-open	0.74/0.79	1.39/1.37
mono-ZZ (small cell)	1.30/1.46	1.90/1.89
mono-ZZ (large cell)	1.37/1.53	1.86/1.84

Validation of minimum

We validate the minimum structure of the detached precursors solvated in liquid Cu below the graphene ribbon using different DFT calculations to exclude an artifact of the MTP potential or the reference DFT-dispersion method. First, two representative configurations are taken from the test dataset, and the energies and forces are computed by PBE+D3 and PBE+MBD with and without spin polarization. The comparison of D3 and MBD is performed to investigate robustness in terms of dispersion interaction, and the inclusion of spin is performed to test for sensitivity of the description of a carbon monomer and its interaction of dangling bonds with the liquid metal. For both dispersion methods and the included spin, the energy differences are within 0.1 meV per atom, while the maximum force differences are less than 0.001 eV/Å, as shown in Figure S20. Furthermore, a comparison between the trained MTP and spin-polarized PBE+MBD is performed. The energy RMSE is 4.5 meV per atom, and the force RMSE is 0.098 eV/Å, as shown in Figure S21. The high agreement indicates spin polarization does not play an important role in this free-energy minimum, and the choice of the dispersion correction does not

affect the minimum either. We further test the localization of the minimum position with respect to the size of the graphene ribbon for mono-ZZ. Using a larger ribbon with a length of ~38 Å ($C_{577}Cu_{1754}$) (instead of ~ 24 Å), we perform an MD simulation for 4 ns, introducing a bias potential that ensures a minimum distance between the monomer and the graphene ribbon larger than 2.5 Å. The monomer delocalizes even over the larger ribbon, indicating a full delocalization of the reactants under the graphene sheet (Figure S22). We note that the delocalization beyond a minimal flake size (see also discussion above) does not lead to a difference in the attachment barrier in our US due to the localization in the CV. Only a significantly smaller ribbon without any stabilization via delocalization (see Figure S15) leads to lower attachment barriers.

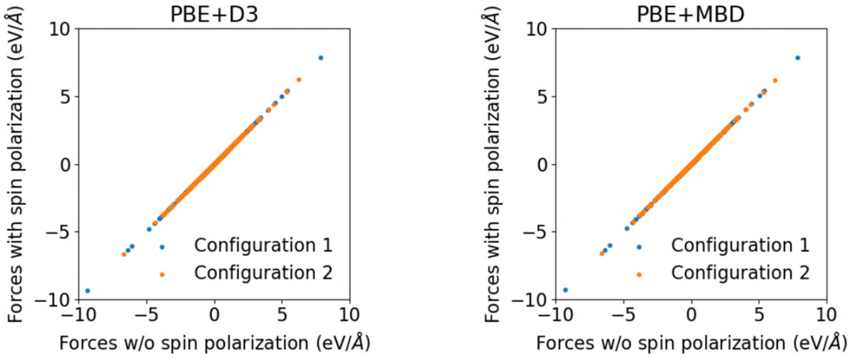

Figure S20. Comparison of forces computed via different DFT settings for two representative configurations of the minimum structure of the detached state in mono-ZZ. We compute forces with and without spin polarization by PBE+D3(left) and PBE+MBD(right) functionals, respectively.

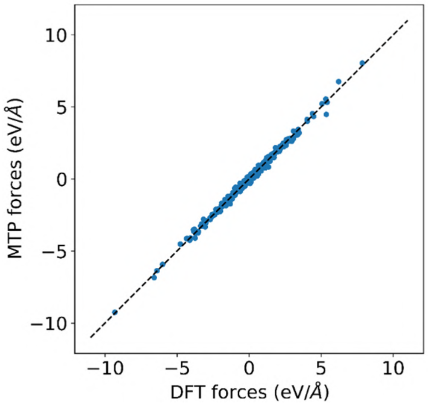

Figure S21. Comparison of forces predicted by MTP and spin-polarized DFT on two representative configurations of the minimum structure of the detached state in mono-ZZ.

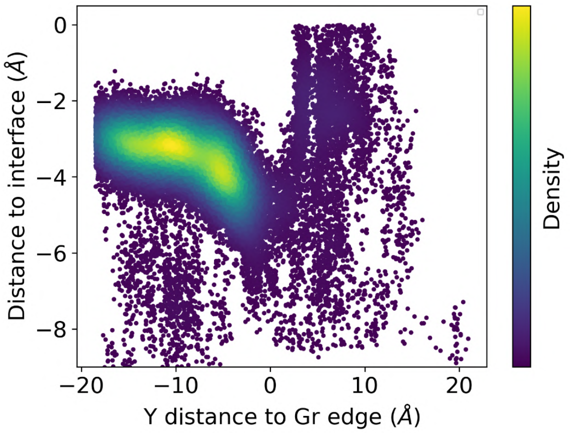

Figure S22. Sampling density for the biased mono-ZZ MD simulation with a large graphene ribbon (see text) projected onto relative coordinates in cartesian space. The horizontal axis describes the minimum distance between carbon species and the graphene edge. Negative values mean that the carbon species is under the graphene ribbon, while positive values mean the monomer is far from the graphene ribbon. The vertical axis describes the distance between the interface and carbon species. Negative values mean the carbon species is immersed in the liquid Cu. The colormap indicates the sampling density, where the highest density indicates the minimum location corresponding to the delocalized state under the graphene sheet.

Microkinetic model of competing carbon monomer and dimer attachment

The apparent growth and etching barriers which we measure in experiment, are determined by the combined attachment and detachment processes of the considered monomer and dimer reactants. The latter depend on the individual reaction barriers as well as the reactant concentrations. While our US simulations of the monomer and dimer attachment processes yield the required attachment and detachment barriers, the reactant concentrations are not easily determined. This is due to the fact that the dimer association barrier on liquid Cu is high and has to be overcome to form a carbon dimer from a monomer that is originally formed after the dissociative adsorption of a methane precursor. This intermediate process leads to a steady-state equilibrium that defines the carbon monomer and dimer population. This steady-state population can significantly deviate from the easily determined equilibrium population, which derives from the free-energy difference between monomer and dimer of ~ 0.3 eV, leading to a strongly dominating dimer population (compare Table S3). An analysis of the influence of the kinetic steady-state equilibrium is thus necessary in order to determine the reactant concentrations and to estimate their effective (combined) attachment barrier. Note that such an analysis has been deemed obsolete for the case of graphene growth on solid Cu, where high reaction barrier differences allowed for the interpretation of a primarily attaching dimer.[30]

In order to gain an understanding of the expected steady-state equilibrium, we formulate a simple mean field microkinetic model to estimate specifically the attachment process competition. Our model includes the dissociative adsorption of methane $\mathrm{CH_4(g)}$ to form a monomer reactant $\mathrm{C^*}$ (1), the dimer $\mathrm{C_2^*}$ association from two monomers (2) and the attachment of a monomer (3) and dimer (4) to a graphene edge $\mathrm{Gr(g)}$:

$$\mathrm{CH_4}(g) + * \rightarrow \mathrm{C^*} + 2\mathrm{H_2} \qquad (1)$$

$$\mathrm{C^*} + \mathrm{C^*} \rightleftharpoons \mathrm{C_2^*} + * \qquad (2)$$

$$\mathrm{C^*} \rightleftharpoons \mathrm{Gr(g)} + * \qquad (3)$$

$$\mathrm{C_2^*} \rightleftharpoons \mathrm{Gr(g)} + * \qquad (4)$$

where * represents a free site, and we assume Gr(g) as a thermodynamic sink (noted for convention as a gas). In this model, we do not discriminate between species on the Cu surface or in the bulk since transitions between these states are already incorporated in our computed reaction barriers (compare Fig. S15, S17, and S21). Thus, we refer to populations θ_i (of species i) instead of coverages in comparison to a surface model. Since a mean-field model assumes perfect mixing and an abstract site * circumvents any complex topological considerations, the working system's inhomogeneity, which is composed of domains of liquid catalyst surface and graphene flakes, is ignored in our estimates. Said inhomogeneity typically requires reactant diffusion to the graphene flakes, resulting in concentration gradients and possibly transport limitations. The latter are, therefore, not present in our model, and their disregard likely leads to an overestimation of the probability of the attachment processes that usually only occur at domain boundaries as compared to the dimer association and methane adsorption. For this reason, we introduce an unknown scaling factor $f_{\mathrm{attachment}}$ which scales the probability of reactant attachment as a free parameter. Further, we did not compute the dissociative adsorption of methane in this work but included this complex process as a free parameter in the form of an effective free energy barrier $\Delta G^{\ddagger}_{\mathrm{CH_4-ads}}$ in our model. We choose the dissociative adsorption of methane as an irreversible process in order to avoid an additional estimated barrier for associative desorption. The latter would introduce more complexity due to the influence of hydrogen gas and unnecessarily convolute our simplified analysis. Using the energies predicted for associative desorption on solid Cu(111),[8] we ensured that including reversible methane adsorption in our model only leads to minor quantitative deviations (which are only found for small values of $\Delta G^{\ddagger}_{\mathrm{CH_4-ads}}$). For all remaining (non-estimated) processes between intermediates $i \rightarrow j$, we employ the corrected reaction barriers $\Delta G^{\ddagger}_{ij}$ from Table S3 for the processes at the ZZ-edge. We compute rate constants as $k_{ij} = \frac{k_B T}{h} e^{\frac{-\Delta G^{\ddagger}_{ij}}{k_B T}}$ where k_B is the Boltzmann constant, T the temperature, and h Planck's constant.[31] In our simulations, we use the experimental temperature range (1380–1450 K) and methane partial pressure range (3.16×10^{-5}–3.95×10^{-4} atm at 1.58×10^{-2} atm hydrogen pressure) as the methane activity. We solve the system of time t dependent coupled differential equations (1–4) for the steady-state condition $\frac{d\theta_i}{dt} = 0$ using the CatMAP software package[32].

In Fig. S23, we display the influence of the free parameters $\Delta G^{\ddagger}_{\mathrm{CH_4-ads}}$ and $f_{\mathrm{attachment}}$ on the reaction mechanism in our microkinetic model. To this end, we compare the attachment rates, the reactant coverages and the degree of rate control (DRC, X_i) according to the definition of Campbell *et al.*[33] of the different reaction barriers (i = $\Delta G^{\ddagger}_{\mathrm{CH_4-ads}}$, $\Delta G^{\ddagger}_{\mathrm{mono-ZZ}}$, and $\Delta G^{\ddagger}_{\mathrm{dimer-ZZ}}$). The range of the free parameter $f_{\mathrm{attachment}}$ is chosen proportional to the ratio of flake circumference to free catalyst area (see Figures 2 and 3 in the main text), which we estimate as 1-0.001, corresponding to an attachment slowdown of 1-1000. With decreasing $f_{\mathrm{attachment}}$, we find a moderately increasing rate of dimer attachment which is accompanied by a shift in DRC (left panels in Figure S23) since the thermodynamically favored conversion from monomer to dimer is accelerated within the kinetic steady-state equilibrium. At the same time, a decreasing $f_{\mathrm{attachment}}$ leads to an increased reactant population, which favors an attachment rate control (right panels in Figure S23). In comparison, we increase $\Delta G^{\ddagger}_{\mathrm{CH_4-ads}}$ from zero within a reasonable range and find that it strongly enforces a dominance of the monomer attachment process. This trend can be explained via the total coverage where a low $\Delta G^{\ddagger}_{\mathrm{CH_4-ads}}$(< 0.8-1.5 eV) leads to large reactant populations $\theta_{\mathrm{monomer}}$ + θ_{dimer} > 10% and higher barriers to negligible populations $\theta_{\mathrm{monomer}}$ + θ_{dimer} << 1% (shown in the top right panel of Figure S23). The large populations promote dimer formation and, thus, dimer attachment. We can derive the observed relationship analytically when assuming the attachment barriers are irreversible (which is a fair approximation given them 0.3 eV

higher than the attachment barriers). It follows for the steady state a balance of equation (2) and (4) as $2 \cdot k_{mono \to dimer} \cdot \theta_{mono}^2 \approx k_{dimer-att} \cdot \theta_{dimer}$. We can express the attachment ratio as $\frac{r_{mono-att}}{r_{dimer-att}} = \frac{k_{mono-att} \cdot \theta_{mono}}{k_{dimer-att} \cdot \theta_{dimer}} = \frac{k_{mono-att}}{2 \cdot k_{mono \to dimer} \cdot \theta_{mono}^2}$ which reveals a power law dependence favoring monomer attachment with decreasing monomer coverage which decreases with increasing $\Delta G^{\ddagger}_{\mathrm{CH4-ads}}$. The DRC $X_{\Delta G^{\ddagger}_{\mathrm{CH_4-ads}}}$ changes from minorly to majorly rate-determining when $\Delta G^{\ddagger}_{\mathrm{CH_4-ads}}$ is > 1.0/2.0 eV (in dependence on $f_{\mathrm{attachment}}$). Within this mechanistic transition range, the DRC gradually changes, indicating a complex mixed mechanism. It is important to note, that within the screened parameter space, the dimer attachment rate dominates at most by about an order of magnitude (< 90%). This means that the dimer is at most moderately dominating; otherwise, the reactants will be of prevalent mixed populations and with increasing $\Delta G^{\ddagger}_{\mathrm{CH_4-ads}}$ monomer attachment is dominant. We finally note that the influence of the methane pressure $P_{\mathrm{CH_4}}$ is inversely related to $\Delta G^{\ddagger}_{\mathrm{CH_4-ads}}$ (but yields a linear instead of an exponential trend).

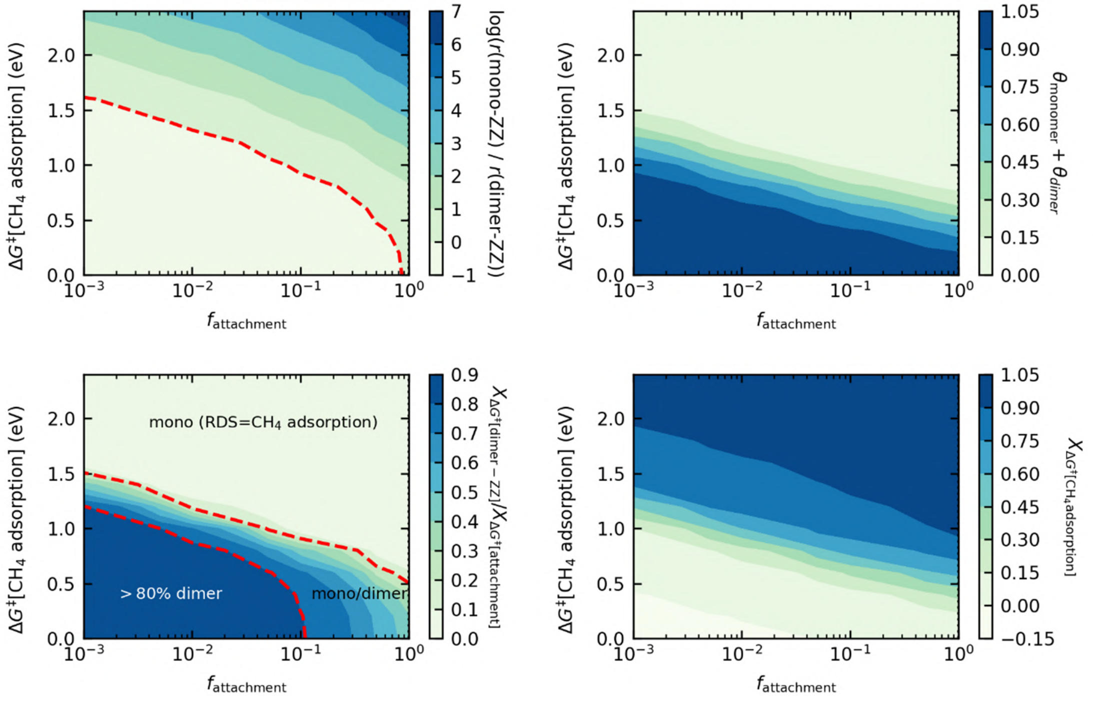

Figure S23. Mechanistic analysis of the rate competition between monomer and dimer and methane activation. (top left) Dependence of the steady-state attachment rate competition between monomer and dimer (top right) the total reactant population $\theta_{mononer} + \theta_{dimer}$, (bottom left) degree-of-rate-control (DRC) of the attachment competition of monomer and dimer, and (bottom right) DRC of $\Delta G^{\ddagger}_{CH_4-ads}$ in dependence on the free parameters $\Delta G^{\ddagger}_{CH_4-ads}$ and $f_{attachment}$ at T=1380 K and p_{CH_4} = 0.032 mbar. Note, that we indicate the attachment rate competition in the panel (top left) via the logarithm of the ratio of the monomer and dimer attachment rates (colormap), where positive values indicate a dominating monomer attachment, negative values a dominating dimer attachment, and the red dashed contour line (ratio = 0) indicates equal attachment rates. Similarly, the DRC of the same competition (bottom left) is expressed as the DRC fraction where the red dashed contour lines indicate the different mechanistic attachment regimes.

In order to relate the simulation results to our experimental data, we evaluate the apparent activation energy $\Delta E^{\ddagger}_{\mathrm{apparent}}$ of our microkinetic model for the competitive monomer/dimer attachment. The apparent attachment barrier is obtained from the temperature dependence of the total attachment rate via the Arrhenius relation. Analog to our experiments, we perform this analysis in dependence on the free parameters $\Delta G^{\ddagger}_{\mathrm{CH_4-ads}}$ and $f_{\mathrm{attachment}}$ as well as for different CH_4 partial pressures, as shown in Figure S24. As marked in the figure, we find an $\Delta E^{\ddagger}_{\mathrm{apparent}}$ within the experimental confidence interval of 1.9±0.3 eV only at high $\Delta G^{\ddagger}_{\mathrm{CH_4-ads}}$ where methane adsorption is the major RDS ($X_{\Delta G^{\ddagger}_{\mathrm{CH_4-ads}}} > 0.75$), which is independent of $f_{\mathrm{attachment}}$ or methane pressure. Here, the $\Delta E^{\ddagger}_{\mathrm{apparent}}$ derives for the most part from the methane adsorption/activation and is only minorly affected by the attachment, proportional to the relative X_i, which extends between 0-25 % depending on the unknown $f_{\mathrm{attachment}}$.

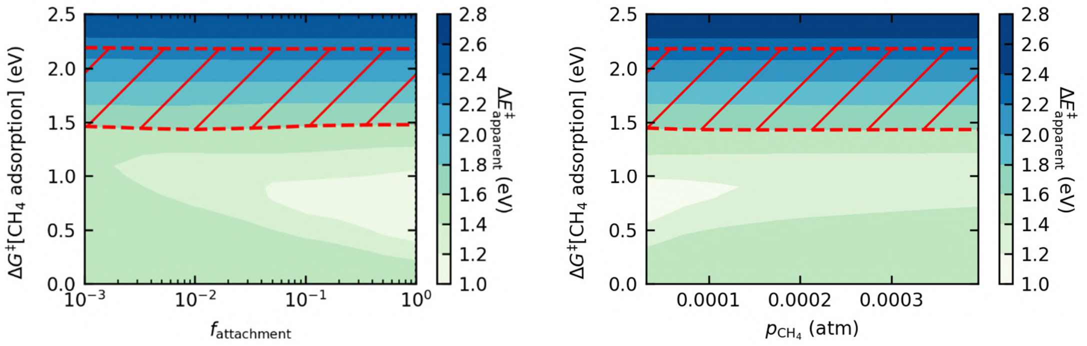

Figure S24. Apparent activation energy ($\Delta E^{\ddagger}_{apparent}$) obtained from the steady-state solution of the mean-field microkinetic model in dependence on different free parameters parameters $\Delta G^{\ddagger}_{CH_4-ads}$ against (left) $f_{attachment}$ at p_{CH_4}= 0.032 mbar and (right) against p_{CH_4} at $f_{attachment}$=0.01 (which we deem to give the best agreement with experiment). We evaluate the apparent activation energy (see colormap) via an Arrhenius relation in the experimental temperature range (1380–1450 K). In red, we mark the parameter regimes where the microkinetic model yields $\Delta E^{\ddagger}_{apparent}$ in agreement with experiment.

Following our analysis of $\Delta E^{\ddagger}_{\mathrm{apparent}}$ and its dependence on $\Delta G^{\ddagger}_{\mathrm{CH_4-ads}}$ and $f_{\mathrm{attachment}}$, we conclude that the graphene growth mechanism is most likely of mixed influence (e.g., 80% methane adsorption/activation and 20% attachment limitation). The major influence of the methane adsorption/activation is in line with the relatively high $\Delta G^{\ddagger}_{\mathrm{CH_4-ads}}$ of 2.3–2.5 eV estimated for solid Cu.[8] Considering the experimentally observed flake shapes and the observed growth rate dependence on flake radius, we do not assume methane adsorption as the sole RDS. The latter, as the sole RDS, would lead to diminishing reactant populations (see Figure S23), which would yield constant areal growth rates and would typically lead to predominantly non-compact/ concave dodecagon flake shapes.[11] Thus, we estimate the value of $f_{\mathrm{attachment}} \leq 0.01$.

On a final note, we highlight that our microkinetic model relies on a number of approximations to simplify the complex chemistry at play. Highlighted by our free-energy simulations, a complex free-energy landscape with surprising local minima (i.e., reactants delocalized under the graphene flake) and self-healing processes can hardly be captured with all its implications by a simple correction like the free parameter $f_{\mathrm{attachment}}$. Similarly, the decisive role of H_2 has been ignored in our model and could yet add another layer of complexity. In that respect, we note that our analysis in Figure S24 still yields $\Delta E^{\ddagger}_{\mathrm{apparent}}$ of 1.4–1.6 eV at $\Delta G^{\ddagger}_{\mathrm{CH_4-ads}} = 0$ (as an intrinsic $\Delta E^{\ddagger}_{\mathrm{attachment}}$ recovering the computed attachment barrier range) that is still relatively close to the experimental confidence interval. Hence, our analysis cannot fully exclude a purely attachment-limited mechanism from a theoretical standpoint.

However, the observed flake shape dependence indicates our estimates and a mixed mechanism as very likely.

References

(1) Tetlow, H.; Posthuma de Boer, J.; Ford, I. J.; Vvedensky, D. D.; Coraux, J.; Kantorovich, L. Growth of Epitaxial Graphene: Theory and Experiment. *Phys. Rep.* **2014**, *542*, 195–295.

(2) Au, C.; Ng, C.; Liao, M. Methane Dissociation and Syngas Formation on Ru, Os, Rh, Ir, Pd, Pt, Cu, Ag, and Au: A Theoretical Study. *J. Catal.* **1999**, *185*, 12–22.

(3) Gan, L.; Luo, Z. Turning off Hydrogen to Realize Seeded Growth of Subcentimeter Single-Crystal Graphene Grains on Copper. *ACS Nano* **2013**, *7*, 9480–9488.

(4) Schwoebel, R. L.; Shipsey, E. J. Step Motion on Crystal Surfaces. *J. Appl. Phys.* **1966**, *37*, 3682–3686.

(5) Vlassiouk, I.; Regmi, M.; Fulvio, P.; Dai, S.; Datskos, P.; Eres, G.; Smirnov, S. Role of Hydrogen in Chemical Vapor Deposition Growth of Large Single-Crystal Graphene. *ACS Nano* **2011**, *5*, 6069–6076.

(6) Losurdo, M.; Giangregorio, M. M.; Capezzuto, P.; Bruno, G. Graphene CVD Growth on Copper and Nickel: Role of Hydrogen in Kinetics and Structure. *Phys. Chem. Chem. Phys.* **2011**, *13*, 20836–20843.

(7) Wu, B.; Geng, D.; Xu, Z.; Guo, Y.; Huang, L.; Xue, Y.; Chen, J.; Yu, G.; Liu, Y. Self-Organized Graphene Crystal Patterns. *NPG Asia Mater.* **2013**, *5*, e36.

(8) Tsakonas, C.; Manikas, A. C.; Andersen, M.; Dimitropoulos, M.; Reuter, K.; Galiotis, C. In Situ Kinetic Studies of CVD Graphene Growth by Reflection Spectroscopy. *Chem. Eng. J.* **2021**, *421*, 129434.

(9) Kalbac, M.; Frank, O.; Kavan, L. The Control of Graphene Double-Layer Formation in Copper-Catalyzed Chemical Vapor Deposition. *Carbon N. Y.* **2012**, *50*, 3682–3687.

(10) Zhang, Y.; Zhang, L.; Kim, P.; Ge, M.; Li, Z.; Zhou, C. Vapor Trapping Growth of Single-Crystalline Graphene Flowers: Synthesis, Morphology, and Electronic Properties. *Nano Lett.* **2012**, *12*, 2810–2816.

(11) Meca, E.; Lowengrub, J.; Kim, H.; Mattevi, C.; Shenoy, V. B. Epitaxial Graphene Growth and Shape Dynamics on Copper: Phase-Field Modeling and Experiments. *Nano Lett.* **2013**, *13*, 5692–5697.

(12) Deokar, G.; Avila, J.; Razado-Colambo, I.; Codron, J. L.; Boyaval, C.; Galopin, E.; Asensio, M. C.; Vignaud, D. Towards High Quality CVD Graphene Growth and Transfer. *Carbon N. Y.* **2015**, *89*, 82–92.

(13) Lin, J.; Guo, L.; Huang, Q.; Jia, Y.; Li, K.; Lai, X.; Chen, X. Anharmonic Phonon Effects in Raman Spectra of Unsupported Vertical Graphene Sheets. *Phys. Rev. B - Condens. Matter Mater. Phys.* **2011**, *83*, 125430.

(14) Jankowski, M.; Saedi, M.; La Porta, F.; Manikas, A. C.; Tsakonas, C.; Cingolani, J. S.; Andersen, M.; De Voogd, M.; Van Baarle, G. J. C.; Reuter, K.; et al. Real-Time Multiscale Monitoring and Tailoring of Graphene Growth on Liquid Copper. *ACS Nano* **2021**, *15*, 9638–9648.

(15) Kim, H.; Mattevi, C.; Calvo, M. R.; Oberg, J. C.; Artiglia, L.; Agnoli, S.; Hirjibehedin, C. F.; Chhowalla, M.; Saiz, E. Activation Energy Paths for Graphene Nucleation and Growth on Cu.

ACS Nano **2012**, *6*, 3614–3623.

(16) Celebi, K.; Cole, M. T.; Choi, J. W.; Wyczisk, F.; Legagneux, P.; Rupesinghe, N.; Robertson, J.; Teo, K. B. K.; Park, H. G. Evolutionary Kinetics of Graphene Formation on Copper. *Nano Lett.* **2013**, *13*, 967–974.

(17) Blum, V.; Gehrke, R.; Hanke, F.; Havu, P.; Havu, V.; Ren, X.; Reuter, K.; Scheffler, M. Ab Initio Molecular Simulations with Numeric Atom-Centered Orbitals. *Comput. Phys. Commun.* **2009**, *180*, 2175–2196.

(18) Perdew, J. P.; Burke, K.; Ernzerhof, M. Generalized Gradient Approximation Made Simple. *Phys. Rev. Lett.* **1996**, *77*, 3865–3868.

(19) Hermann, J.; Tkatchenko, A. Density Functional Model for van Der Waals Interactions: Unifying Many-Body Atomic Approaches with Nonlocal Functionals. *Phys. Rev. Lett.* **2020**, *124*, 146401.

(20) Shapeev, A. V. Moment Tensor Potentials: A Class of Systematically Improvable Interatomic Potentials. *Multiscale Model. Simul.* **2016**, *14*, 1153–1173.

(21) Novikov, I. S.; Gubaev, K.; Podryabinkin, E. V; Shapeev, A. V. The MLIP Package: Moment Tensor Potentials with MPI and Active Learning. *Mach. Learn. Sci. Technol.* **2021**, *2*, 025002.

(22) Gao, H.; Belova, V.; Porta, F. La; Cingolani, J. S.; Andersen, M.; Saedi, M.; Konovalov, O. V; Jankowski, M.; Heenen, H. H.; Groot, I. M. N.; et al. Graphene at Liquid Copper Catalysts: Atomic-Scale Agreement of Experimental and First-Principles Adsorption Height. *Adv. Sci.* **2022**, *9*, 2204684.

(23) Gelžinytė, E.; Wengert, S.; Stenczel, T. K.; Heenen, H. H.; Reuter, K.; Csányi, G.; Bernstein, N. Wfl Python Toolkit for Creating Machine Learning Interatomic Potentials and Related Atomistic Simulation Workflows. *J. Chem. Phys.* **2023**, *159*, 124801.

(24) Valsson, O.; Parrinello, M. Variational Approach to Enhanced Sampling and Free Energy Calculations. *Phys. Rev. Lett.* **2014**, *113*, 90601.

(25) Tribello, G. A.; Bonomi, M.; Branduardi, D.; Camilloni, C.; Bussi, G. PLUMED 2: New Feathers for an Old Bird. *Comput. Phys. Commun.* **2014**, *185*, 604–613.

(26) Thompson, A. P.; Aktulga, H. M.; Berger, R.; Bolintineanu, D. S.; Brown, W. M.; Crozier, P. S.; in 't Veld, P. J.; Kohlmeyer, A.; Moore, S. G.; Nguyen, T. D.; et al. LAMMPS - a Flexible Simulation Tool for Particle-Based Materials Modeling at the Atomic, Meso, and Continuum Scales. *Comput. Phys. Commun.* **2022**, *271*, 108171.

(27) Andersen, M.; Cingolani, J. S.; Reuter, K. Ab Initio Thermodynamics of Hydrocarbons Relevant to Graphene Growth at Solid and Liquid Cu Surfaces. *J. Phys. Chem. C* **2019**, *123* (36), 22299–22310.

(28) Tuckerman, M. E.; Alejandre, J.; López-Rendón, R.; Jochim, A. L.; Martyna, G. J. A Liouville-Operator Derived Measure-Preserving Integrator for Molecular Dynamics Simulations in the Isothermal-Isobaric Ensemble. *J. Phys. A. Math. Gen.* **2006**, *39*, 5629–5651.

(29) Dietschreit, J. C. B.; Diestler, D. J.; Hulm, A.; Ochsenfeld, C.; Gómez-Bombarelli, R. From Free-Energy Profiles to Activation Free Energies. *J. Chem. Phys.* **2022**, *157*, 084113.

(30) Wu, P.; Zhang, Y.; Cui, P.; Li, Z.; Yang, J.; Zhang, Z. Carbon Dimers as the Dominant Feeding Species in Epitaxial Growth and Morphological Phase Transition of Graphene on Different Cu Substrates. *Phys. Rev. Lett.* **2015**, *114*, 216102.

(31) Nørskov, J. K.; Studt, F.; Abild-Pedersen, F.; Bligaard, T. *Fundamental Concepts in Heterogeneous Catalysis*; John Wiley & Sons, 2014.

(32) Medford, A. J.; Shi, C.; Hoffmann, M. J.; Lausche, A. C.; Fitzgibbon, S. R.; Bligaard, T.; Nørskov, J. K. CatMAP: A Software Package for Descriptor-Based Microkinetic Mapping of Catalytic Trends. *Catal. Letters* **2015**, *145*, 794–807.

(33) Stegelmann, C.; Andreasen, A.; Campbell, C. T. Degree of Rate Control: How Much the Energies of Intermediates and Transition States Control Rates. *J. Am. Chem. Soc.* **2009**, *131*, 13563.
